# Improving error reporting at the ICU

**DOI:** 10.1186/2197-425X-3-S1-A68

**Published:** 2015-10-01

**Authors:** EE Rouw, T van Galen

**Affiliations:** RN, VU University Medical Center, Intensive Care, Amsterdam, Netherlands; ICU Nursing Staff Manager, VU University Medical Center, Amsterdam, Netherlands

## Introduction

A significant number of dangerous incidents occur at the ICU on a daily basis[1]. These incidents or (near) errors are suitable to identify weaknesses in care systems. Therefore, it is important that all incidents are reported and analysed[2]. At the ICU at VU University medical center (VUmc), in 2009 less then 100 incidents were reported and throughput and analysis were long. Also little improvement measures were formulated and feedback to the team was minimal. To increase the number of reportings, improvement measures were formulated as regard to culture and structure.

## Methods

Handling ICU error reporting is conducted by a monthly meeting, multidisciplinary committee.

Culture adjustments made: promoting an accessible and blaim free[3] reporting culture, learning from errors is the key principle and creating constant risk awareness and attention for improvement measures in team meetings and thematic consultation. With quarterly reports and whiteboard notifications current risks or preventive measures are linked back. The committee acts directly in the ICU environment.

Structure adjustments made: To advance individual processing and handling error reporting an activity matrix was implemented and reported errors are classified by location and specialism. To improve error analysis and increase improvement measures the PRISMA tool was implemented in the monthly workflow.

A specific “reporting button” was developed within the EHR (Metavison) to improve reporting accessibility.

## Results

From 2009 to 2014 is the number of reports has increased from < 100 a year up to > 600 a year. Throughput of semi serious incidents has decreased from 10 weeks to 4. Team feedback indicates more insight of errors reported and improvement measures formulated. The development of the “incident button” has resulted in an accelerated increase in the number of ADE´s reportings: in the first 12 weeks there were as many ADE´s reported compared to the previous 12 months.

## Discussion

To increase error reports at the ICU, improvement measures were succesfully implemented to change an inefficient, barrier creating reporting system. These changes aren't only due to structure adjustments, it requires also a change of culture. At the ICU of VUmc, these improvements resulted in an increased number of error reportings, especially less serious incidents. The incident button in the EHR has generated the most profit due to optimal workflow feasibility and benefits for committee members because reporting within the patient file provides more complete and easier accessible information for analysis.
